# GABPB1-AS1 acts as a tumor suppressor and inhibits non-small cell lung cancer progression by targeting miRNA-566/F-box protein 47

**DOI:** 10.32604/or.2022.025262

**Published:** 2022-11-10

**Authors:** HUALIANG LV, CHANGCHUN LAI, WENQU ZHAO, YIBO SONG

**Affiliations:** 1 Department of Pulmonary and Critical Care Medicine, Maoming People’s Hospital, Maoming, China; 2 Department of Clinical Laboratory, Maoming People’s Hospital, Maoming, China; 3 Department of Pulmonary and Critical Care Medicine, Nanfang Hospital Affiliated to Southern Medical University, Guangzhou, China

**Keywords:** GABPB1-AS1, NSCLC, Progression, miRNA-566, FBXO47

## Abstract

It has been certified that GABPB1-AS1 is aberrantly expressed and plays as a vital role in some kinds of cancers. However, its expression pattern and functions in non-small cell lung cancer (NSCLC) are still largely unknown. This study aims to assess GABPB1-AS1 expression and biological roles in NSCLC. The expression of GABPB1-AS1 was detected in NSCLC specimens and adjacent normal specimens. CCK8 and Transwell assays were performed to evaluate the effects of GABPB1-AS1 on NSCLC cell proliferation, migration and invasion. Bioinformatics tools and luciferase reporter assays were applied to predict and verify GABPB1-AS1’s direct targets. The results revealed that GABPB1-AS1 is sharply reduced in NSCLC specimens and cell lines. CCK8 assays indicated that overexpression of GABPB1-AS1 dramatically reduced NSCLC cell growth, and Transwell assays proved that NSCLC cell migration and invasion were distinctly inhibited by GABPB1-AS1. Exploration of the mechanism uncovered that miRNA-566 (miR-566)/F-box protein 47 (FBXO47) is directly targeted by GABPB1-AS1 in NSCLC. The study demonstrated that GABPB1-AS1 inhibited NSCLC cell proliferation, migration and invasion by targeting miR-566/FBXO47.

## Introduction

Lung cancer is the leading cause of cancer-associated mortality worldwide [1]. Non–small cell lung cancer (NSCLC) accounts for 80%–85% of the newly diagnosed lung cancer cases, and it includes adenocarcinoma, squamous cell carcinoma and adenosquamous cell carcinoma, as well as large cell carcinoma [2]. Even though the diagnostic and therapeutic methods have developed a lot in recent years, the prognosis of NSCLC remains poor [3,4]. Hence, it is critical to explore the molecular mechanisms of NSCLC development and find molecular markers for it.

As a member of the RNA family, non-coding RNAs (ncRNAs) are less translated into protein but exhibit essential functions in organisms [5]. Long non-coding RNAs (lncRNAs), a significant member of the ncRNA family, are non-coding transcripts with more than 200 nucleotides [6]. Most lncRNAs were regarded as non-functional molecules before [7,8]. However, recently, increasing evidence demonstrated that some lncRNAs exhibit crucial roles in numerous physiological and pathological processes [9]. Abnormal lncRNA expression and its potential function in tumorigenesis and progression have been noted in various cancers. Some studies indicated that the pathologic processes and progression of NSCLC could be mediated by lncRNAs [10,11]. For the lncRNA role in NSCLC, lncRNA HOTAIR was reported to be overexpressed in NSCLC tissues and cell lines and could promote NSCLC cell invasion and migration abilities [12]. The level of lncRNA PCGsEM1 was correlated with the advanced TNM stage of NSCLC, and lncRNA PCGsEM1 regulated NSCLC cell proliferation, colony formation and invasion by miR-152-3p [13].

The lncRNA GABPB1-AS1 was first identified by Hidenori et al. using 5′-Bromo-uridine immunoprecipitation chase-deep sequencing analysis in HeLa tet-off cells [14]. This distinct lncRNA was also characterized as a potentially surrogate indicator of chemical stress responses in human induced pluripotent stem cells [15]. For its role in cancer, Kim and colleagues firstly found that GABPB1-AS1 was cancer-associated lncRNA in prostate cancer in the Chinese population and later confirmed as potential prostate cancer biomarker using machine learning techniques in Abedalrhman’s study [16]. Moreover, GABPB1-AS1 was also identified as one of the essential autophagy-associated lncRNAs with prognostic value in glioma and may play a key role in its progression [17]. However, no studies have clarified GABPB1-AS1’s expression and latent functions in NSCLC. In present study, the expression profile and the biological functions of GABPB1-AS1 in NSCLC was investigated. In addition, we explored the underlying mechanism of how GABPB1-AS1 influences NSCLC progression.

## Materials and Methods

### Clinical specimens

50 NSCLC patients undergoing surgery in Maoming People’s Hospital participated in this research. None of these underwent antitumor therapy before NSCLC resection. The NSCLC and adjacent normal specimens were collected after resection and immediately placed in liquid nitrogen. All patients provided informed consent, and the Ethics Committee of Maoming People’s Hospital approved this research (201600120030).

### Cell culture and transfection

Human NSCLC cell lines (A549, SPC-A1, H460 and HCL-H359) and normal pulmonary epithelium cell line HBE were acquired from the American Type Culture Collection (ATCC). Dulbecco’s modified Eagle’s medium (Invitrogen, Carlsbad, CA, USA) was used for cell cultivation. The medium was supplemented with 10% fetal bovine serum (FBS) (HyClone, Logan, UT, USA) and 1% penicillin/streptomycin (Invitrogen, Carlsbad, CA, USA). Following the manufacturer’s protocol, lipofectamine 2000 was attained from Invitrogen (Invitrogen, Carlsbad, CA) and applied for transfection. We purchased plasmids of pcDNA-GABPB1-AS1 (GABPB1-AS1) and pcDNA-control (lnc-ctr) from Invitrogen (Invitrogen, Carlsbad, CA), and miRNA-566 mimic (miR-566) and mimic control (miR-ctr) from GenePharma (Shanghai, China). GABPB1-AS1 siRNA (si-GABPB1-AS1) and the negative siRNA control (si-ctr) were obtained from Ribobio (Guangzhou, China). Cells were subject to the subsequent assays 48 h after transfection.

### Cell counting kit 8 assays

Cells were suspended and counted under a microscope. Briefly, 1.5 × 10^3^ cells were resuspended in 100 µl media and seeded in 96-well plates. We added cell counting kit 8 reagents (CCK8) (Dojindo, Tokyo, Japan) to each plate. After 2-h incubation, the absorbance of NSCLC cells at 465 nm was assessed using a microplate reader.

### Cell migration and invasion assays

NSCLC cell migration and invasion capacities were assessed using Transwell inserts (Corning Costar, MA, USA). For invasion assays, the upper chambers of Transwell inserts were carpeted with Matrigel matrix (BD, NJ, USA). Then, 5 × 10^5^ cells were suspended in 300 µl DMEM and placed into the upper chamber. We filled the lower chamber with 400 µl DMEM, which was replenished with 10% FBS. These two chambers were incubated for 36 h, and invading cells were fixed with methanol and stained with 0.1% crystal violet. The migration assays were similar to the invasion assays, except the upper chamber was not coated with Matrigel matrix. The migrated or invaded cells was calculated under a light microscope in five random fields.

### Real-time quantitative reverse transcription PCR

According to the manufacturer’s protocols, Trizol reagent (Invitrogen, Carlsbad, CA, USA) was employed for the isolation of total RNA from the clinical specimens and cell lines. To detedt RNA expression, real-time quantitative reverse transcription PCR (RT-PCR) was performed using a Biosystems RT-PCR machine. The thermocycling conditions of RT-PCR were 10 min at 95°C; 40 cycles of 30 s at 94°C, 30 s at 58°C, 15 s at 72°C; hold at 4°C. The relative expression of the target gene was assessed using the Ct method, and data were standardized to GAPDH or U6 expression. The primers used in this study are presented in Table 1.

**Table 1:** The specific sequence of primers for qRT-PCR

Name	Sequence (5′-3′)	
primers: GABPB1-AS1	CTGGATGGTCGCTGCTTTTTA (forward)	AGGGGGATG AGTCGTGATTT (reverse)
primers: FBXO47	TTCCAAGACCCTTGGCTCAG (forward)	TTCCAAGACCCTTGGCTCAG (reverse)
primers: GAPDH	GCACCGTCAAGGCTGAGAAC (forward)	TGGTGAAGACGCCAGTGGA (reverse)

### Western blots

The total protein from NSCLC cell lines was extracted using RIPA Reagent (Beyotime, Shanghai, China). Then we detected the protein concentrations using a BCA protein assay kit (Beyotime) following the manufacture’s protocol. Total protein was loaded in sodium dodecyl sulfate polyacrylamide gel electrophoresis and transferred to the polyvinylidene difluoride membrane. The polyvinylidene difluoride membrane was then incubated with primary antibody recombinant human F-box protein 47 (FBXO47) overnight at 4°C. The membranes were washed and incubated with the corresponding second antibody. GAPDH was adopted as internal control, and proteins were visualized in chemoluminiscence (Perkin Elmer, USA).

### Dual-luciferase reporter assays

The wild-type (wt) sequence of GABPB1-AS1, which includes the miR-566 targeting site, and the mutant type (mt) sequence were inserted into the luciferase reporter vector pmirGLO (Promega, Madison, WI, USA). The former reporter vectors and miR-566 mimics or mimic control were co-transfected into cells. Luciferase reporter activities in indicated cells were determined using the Dual-Luciferase Reporter Assay System. Outcomes were normalized to Renilla luciferase activity. The targeting effects between miR-566 and FBOX47 were similar.

### In vivo tumorigenesis tests

For *in vivo* tumorigenesis tests, 6-week nude mice (NU/NU) were obtained from Vital River Inc. (Beijing, China). The 12 nude mice were randomly divided into the control group and the GABPB1-AS1 group. 1 × 10^6^ cells were suspended in 300 μl PBS and injected into the flanks of mice subcutaneously. The tumor volume was measured for three weeks continuously.

### Statistical analysis

We applied SPSS 20.0 statistical software package (SPSS, Chicago, IL, USA) to analyze the data, presented it as the mean ± standard error of the mean. The difference between the two groups was compared using the student’s *t*-test. The survival rate and occurrence rate in enrolled patients were estimated by the Kaplan-Meier method and evaluated by the log-rank test. *p* < 0.05 was considered different significantly.

## Results

### LncRNA GABPB1-AS1 was significantly downregulated in human NSCLC

**Figure 1:**
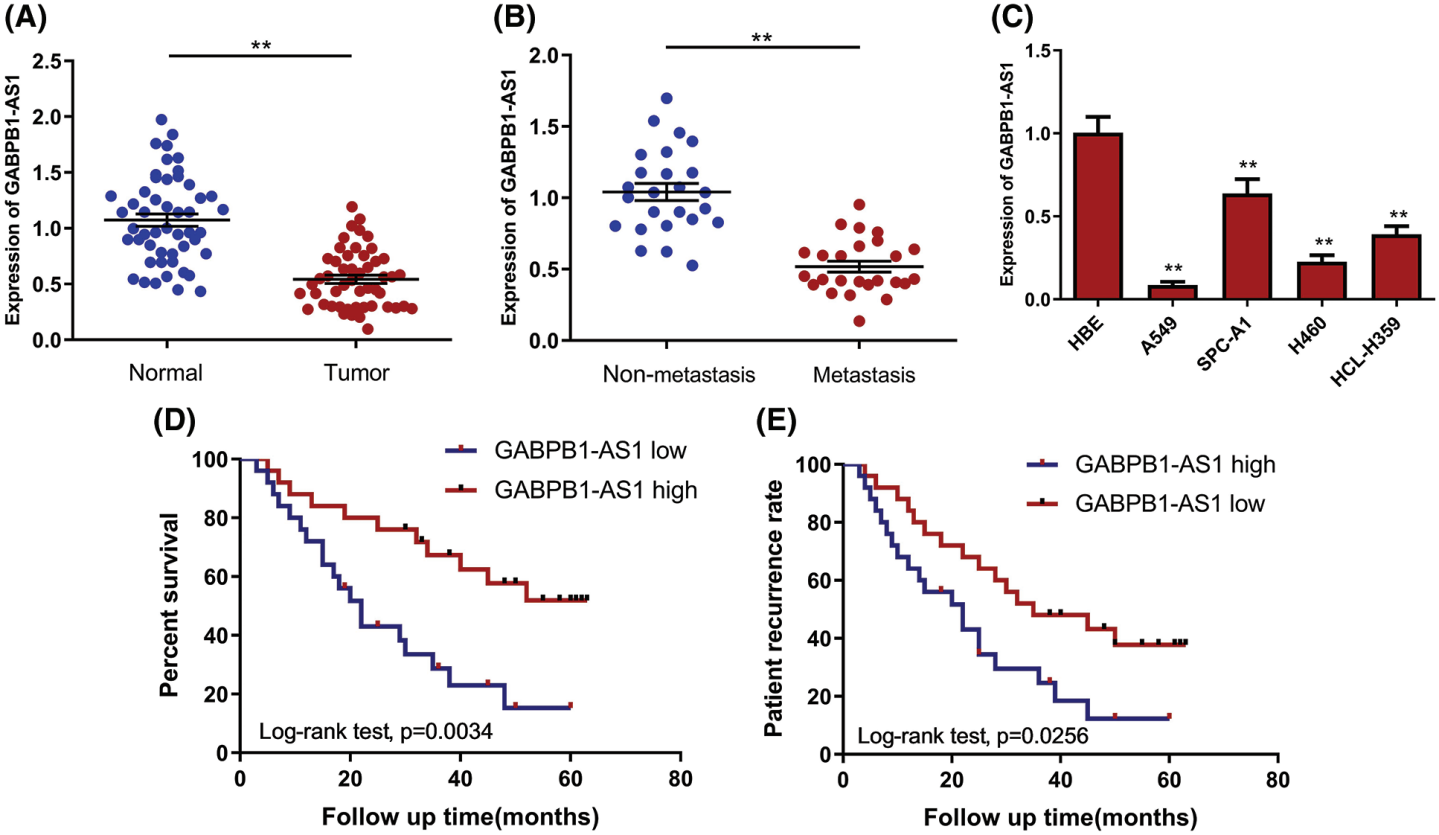
GABPB1-AS1 expression was remarkably reduced in NSCLC. (A) Compared with adjacent normal specimens, GABPB1-AS1 expression was significantly reduced in NSCLC specimens. (B) GABPB1-AS1 expression was dramatically reduced in metastatic NSCLC when compared with non-metastatic NSCLC. (C) Compared with a normal bone cell line, GABPB1-AS1 expression was sharply reduced in NSCLC cell lines. (D and E) The patients with high GABPB1-AS1 showed better survival and recurrence rates. **p < 0.01, all experiments were repeated three times.

To investigate lncRNA GABPB1-AS1’s expressive pattern in human NSCLC, 50 pairs of NSCLC and adjacent normal samples were collected from patients undergoing surgery in Maoming People’s Hospital. We detected the expressions of GABPB1-AS1 by RT-PCR. It was found that GABPB1-AS1 levels were remarkably reduced in NSCLC samples compared with paired normal samples (Fig. 1A). Then, the enrolled patients were classified based on clinical statuses with or without metastasis. Further analyses revealed that GABPB1-AS1 expressions were reduced in patients with metastasis compared with those without metastasis (Fig. 1B). In addition, GABPB1-AS1 was also detected in NSCLC cell lines (A549, SPC-A1, H460 and HCL-H359) and normal pulmonary epithelium cell line HBE. The results illustrated that GABPB1-AS1 was significantly downregulated in NSCLC cell lines compared to the normal pulmonary epithelium cell line (Fig. 1C). Moreover, we explored the relationship between GABPB1-AS1 expression and the prognosis. The enrolled patients were divided into groups based on their GABPB1-AS1 expressive status. It was found that patients with high GABPB1-AS1 showed better survival and recurrence rates (Figs. 1D and 1E).

### NSCLC cell proliferation, migration and invasion are reduced by GABPB1-AS1

**Figure 2:**
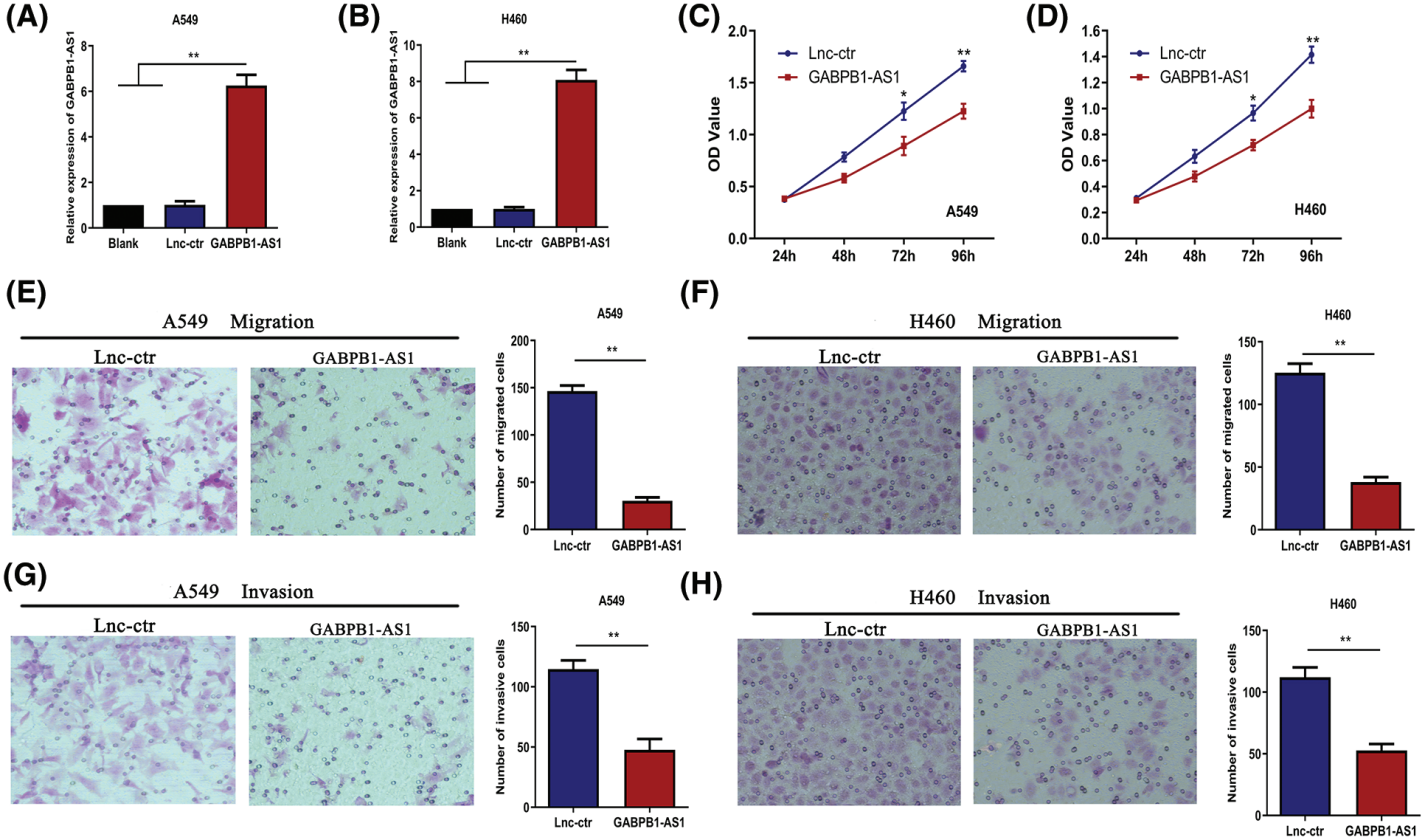
GABPB1-AS1 has suppressive effects on NSCLC cells. (A and B) The transfection efficiency of pc-GABPB1-AS1 was verified in two cell lines. (C and D) GABPB1-AS1 overexpression significantly reduced A549 and H460 cell proliferation. (E and F) GABPB1-AS1 overexpression significantly inhibited A549 cell migration and invasion. (G and H) GABPB1-AS1 overexpression significantly reduced H460 cell migration and invasion. 100× magnification. *p < 0.05, **p < 0.01, all experiments were repeated three times.

We conducted functional analysis in NSCLC cells to explore the biological role of GABPB1-AS1. A549 and H460 cell lines were chosen for functional experiments due to their low endogenous GABPB1-AS1 expression. The pc-GABPB1-AS1 plasmids were transfected into A549 or H460 cells to overexpress GABPB1-AS1. Simultaneously, the control plasmids were transfected as control groups. Quantitative real-time PCR was employed to verify the transfection efficiency, which successfully induced increased expression of GABPB1-AS1 in A549 and H460 cells (Figs. 2A and 2B). CCK8 assays were used to assess the cell proliferative ability. Overexpression of GABPB1-AS1 lncRNA suppressed A549 and H460 cell proliferation (Figs. 2C and 2D). According to Transwell migration and invasion experiments, the migratory and invasive capacities of A549 cells were significantly inhibited by the overexpression of GABPB1-AS1 (Figs. 2E and 2G). Similar results were noted in H460 cells (Figs. 2F and 2H). These data suggested that overexpression of GABPB1-AS1 significantly suppressed NSCLC cell proliferation, migration and invasion.

### GABPB1-AS1 directly targets miRNA-566 in NSCLC cells

**Figure 3:**
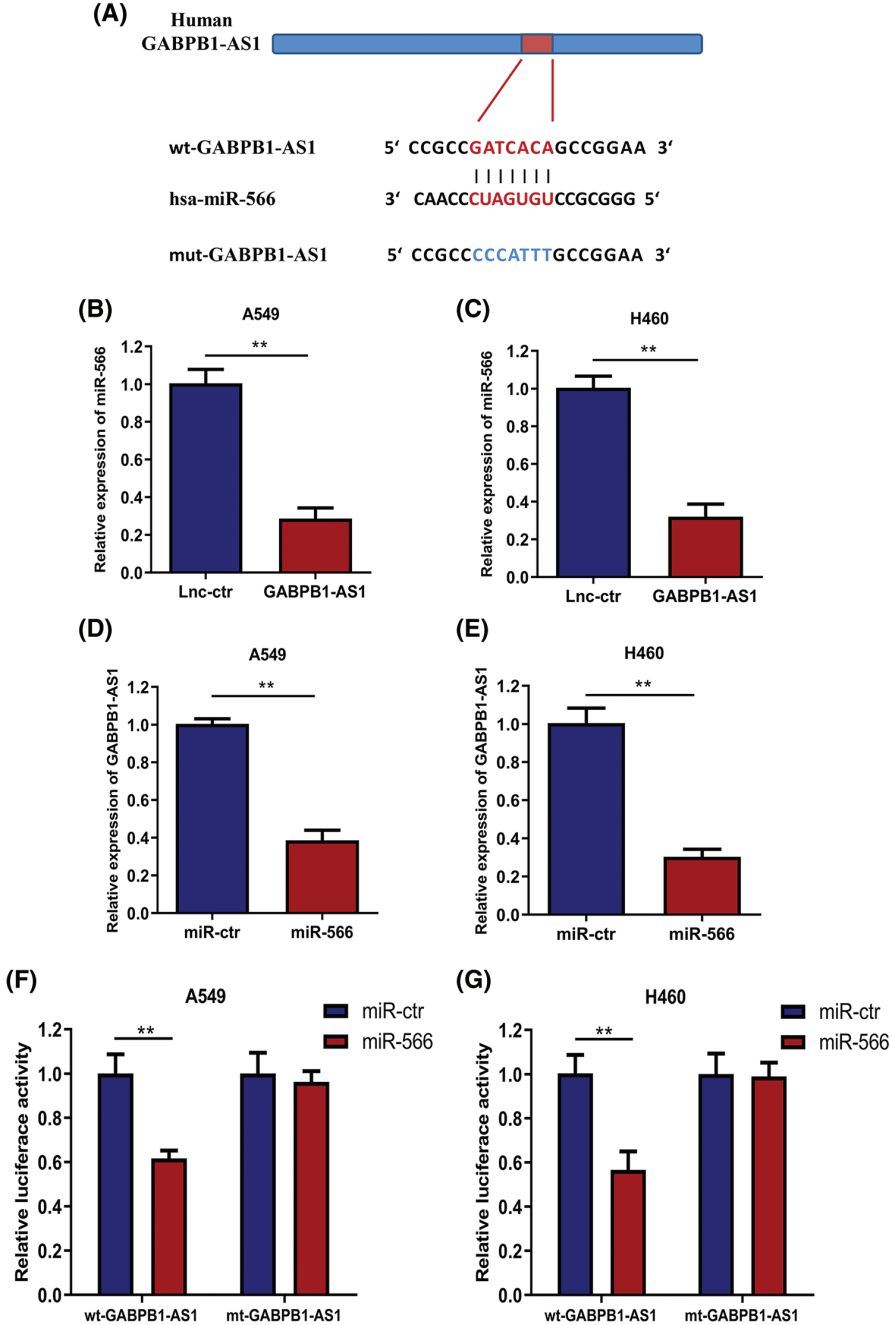
GABPB1-AS1 directly targeted miR-566. (A) GABPB1-AS1 has potential miR-566 binding sequences. (B and C) MiR-566 expressions in A549 and H460 cells were significantly inhibited by the overexpression of GABPB1-AS1. (D and E) Overexpression of GABPB1-AS1 significantly reduced miR-566 levels in A549 and H460 cell. (F) Luciferase reporter assays indicated that miR-566 was targeted directly by GABPB1-AS1 in A549 cell. (G) Luciferase reporter assays revealed that GABPB1-AS1 directly targeted miR-566 in H460 cells. **p < 0.01, all experiments were repeated three times.

Some lncRNAs play essential roles in RNA homeostasis. One of the most critical functions of these functional lncRNAs is acting as competitive endogenous RNA (ceRNA) in regulating networks. To better understand the underlying molecular mechanism by which GABPB1-AS1 affects NSCLC progression, the online bioinformatics tool Starbase 2.0 (http://starbase.sysu.edu.cn) was applied to predict the downstream molecule of GABPB1-AS1. MiRNA-566 (miR-566) was a predicted target of GABPB1-AS1 with a potential binding sequence (Fig. 3A). The expression of miR-566 was first measured in NSCLC cells transfected with GABPB1-AS1. GABPB1-AS1 overexpression significantly suppressed the expression of miR-566 in NSCLC cells (Figs. 3B and 3C). Upon transfection with a miR-566 mimic, we detected the GABPB1-AS1 expression in A549 and H460 cells. GABPB1-AS1 levels were significantly reduced when cells overexpressed miR-566, indicating that lncRNA GABPB1-AS1 might directly inhibit miR-566 (Figs. 3D and 3E). To verify the relation between GABPB1-AS1 and miR-566, plasmids containing a GABPB1-AS1 fragment with the putative miR-566 binding sequence (wt GABPB1-AS1) or the mutant fragment (mt GABPB1-AS1) were co-transfected with miR-566 or miR-ctr into the NSCLC cells. MiR-566 in NSCLC cells with wt-GABPB1-AS1 reduced luciferase activity. Nevertheless, the suppressive effects of miR-566 were abolished in NSCLC cells with mt-GABPB1-AS1 (Figs. 3F and 3G). Therefore, we concluded that lncRNA GABPB1-AS1 could directly bind to miR-566.

### MiR-566 directly targets FBXO47 in NSCLC cells

**Figure 4:**
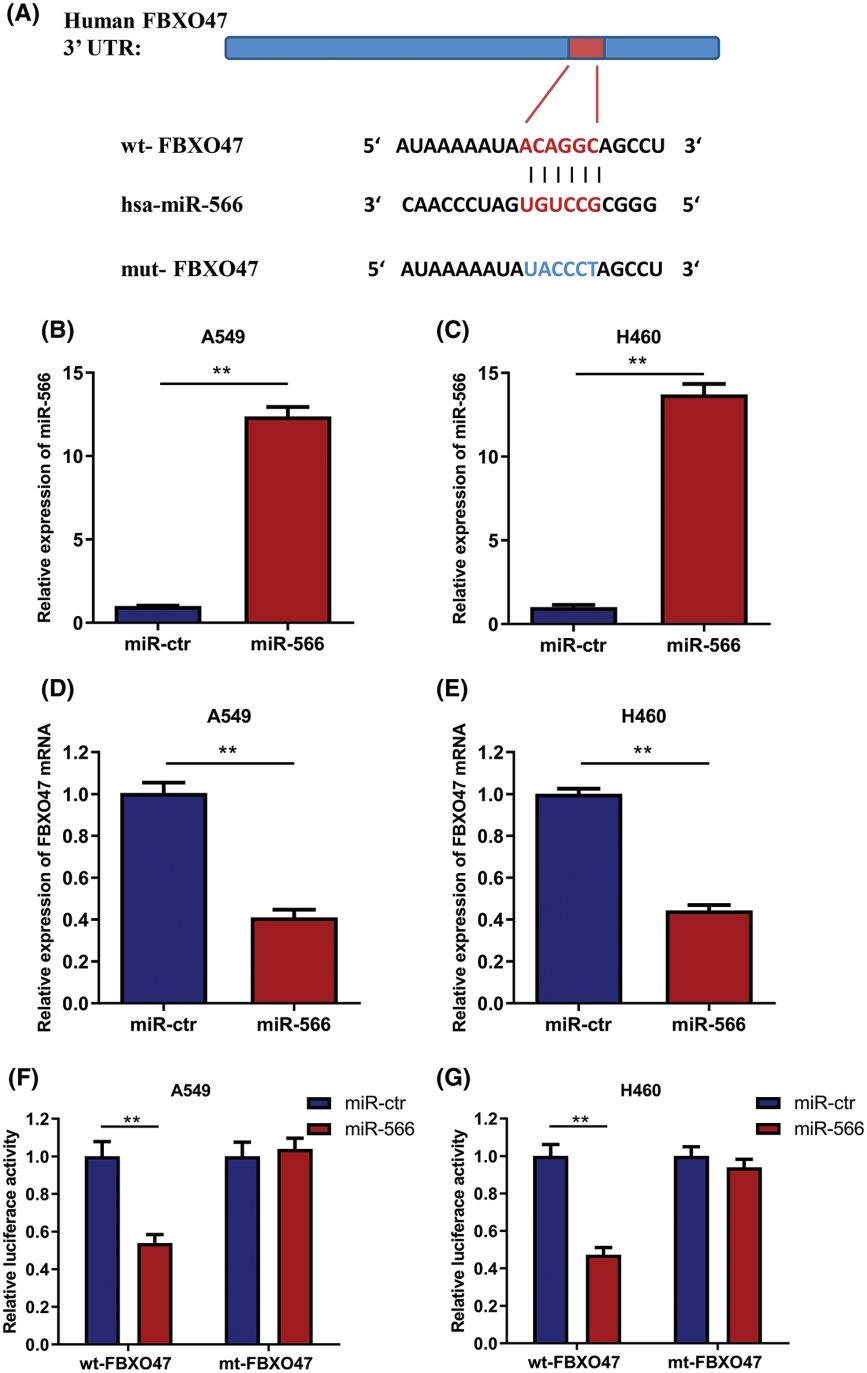
MiR-566 targeted FBXO47 directly in NSCLC cells. (A) FBXO47 contains a potential miR-566 binding sequence. (B and C) The transfection efficiency of miR-566 was calculated in two cell lines. (D and E) FBXO47 expression was inhibited by miR-566 in A549 and H460 cells. (F) Luciferase reporter assay revealed that miR-566 directly targeted FBXO47 in A549 cells. (G) Luciferase reporter assays revealed that FBXO47 was a direct target of GABPB1-AS1 in H460 cells. **p < 0.01, all experiments were repeated three times.

One of the crucial functions of miRNA is that it binds to the 3′UTR of its downstream molecules. Thus, the possible presence of downstream molecules targeted by miR-566 was explored. The online algorithms Targetscan, Starbase and Miranda were used for miR-566 target prediction. FBXO47 was predicted to be a target of miR-566 and contained a potential binding site (Fig. 4A). A549 and H460 cells were then transfected with miR-566 mimic, and the transfection efficiency was verified (Figs. 4B and 4C). FBXO47 mRNA expression was measured in the cells with miR-566 overexpression. Overexpression of miR-566 could remarkably inhibit FBXO47 expression in A549 and H460 cells (Figs. 4D and 4E). Moreover, luciferase reporter assays were conducted to verify the binding effects of miR-566 and FBXO47. Luciferase activity in A549 cells was suppressed by miR-566 when co-transfected with wild-type FBXO47 (wt-FBXO47) but not mutant FBXO47 (mt-FBXO47) (Fig. 4F). The results in H460 cells were consistent with that in A549 cells (Fig. 4G). Therefore, these results indicated that miR-566 directly targeted FBXO47 in NSCLC cells.

### The effects of GABPB1-AS1 in NSCLS cells were mediated by miR-566/FBXO47

**Figure 5:**
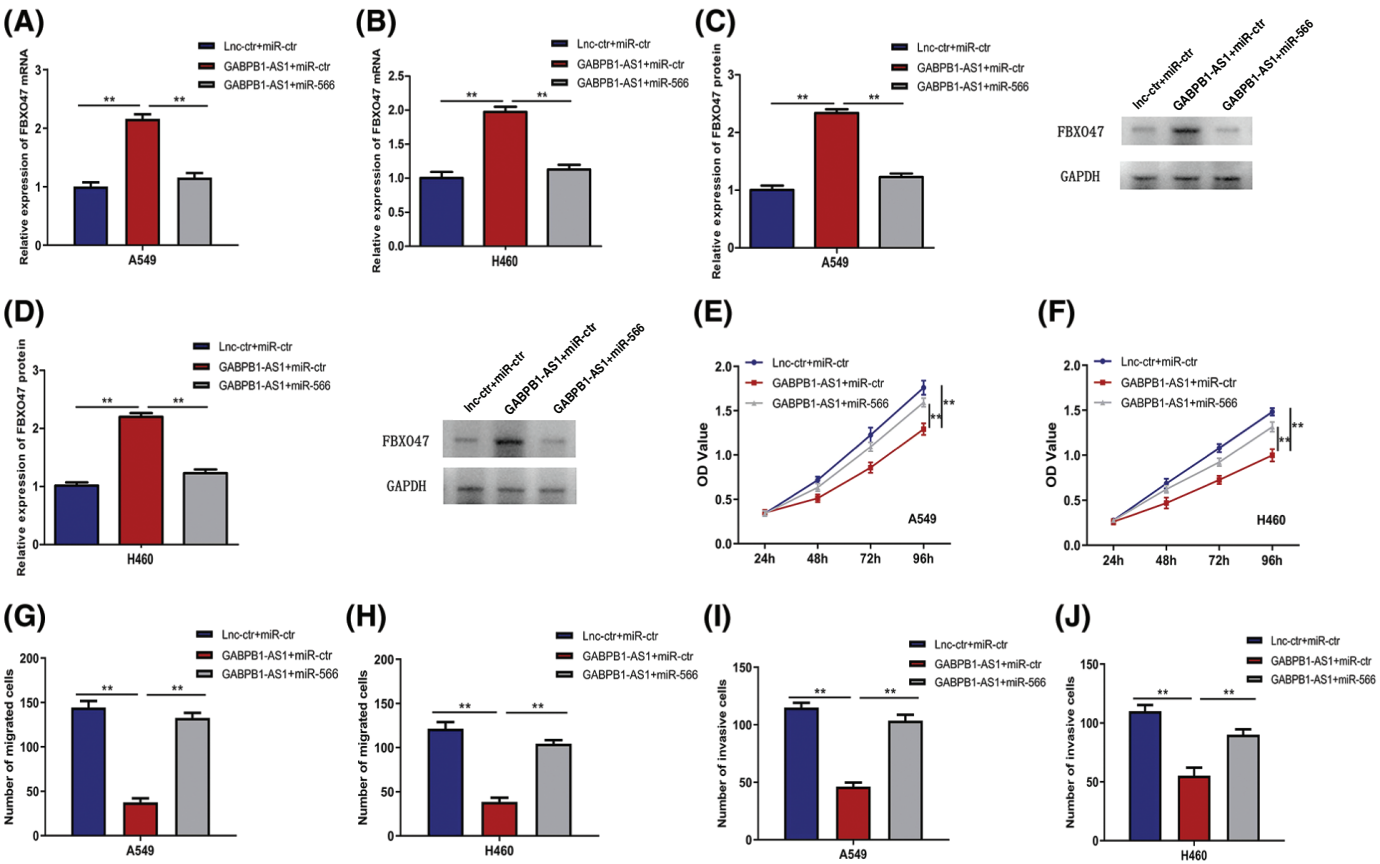
GABPB1-AS1’s effects on NSCLC cells are mediated by miR-566/FBXO47. (A and B) GABPB1-AS1 and miR-566 co-regulate FBXO47’s mRNA expression in A549 and H460 cells. (C and D) Western Blot showed that GABPB1-AS1 and miR-566 co-regulate FBXO47 protein expression in A549 and H460 cells. (E) The suppressive effects of GABPB1-AS1 on A549 cell proliferation were reduced by miR-566. (F) The suppressive effects of GABPB1-AS1 on H460 cell proliferation were reduced by miR-566. (G and H) MiR-566 salvaged the inhibitory effects of GABPB1-AS1 on A549 and H460 cell migration. (I and J) MiR-566 rescued the inhibitory effects of GABPB1-AS1 on A549 and H460 cell invasion. **p < 0.01, all experiments were repeated three times.

To further explore whether the effects of GABPB1-AS1 in NSCLC cells were mediated by miR-566/FBXO47, the GABPB1-AS1 plasmids or their negative control, as well as miR-566 mimics or their negative control, were co-transfected into NSCLC cells, and the phenotype change was evaluated. Cells in the control group were co-transfected with lnc-ctr and miR-ctr, whereas cells in the experimental group were co-transfected with GABPB1-AS1 and miR-ctr. The effects of GABPB1-AS1 were determined. The cells in the rescued group were co-transfected with GABPB1-AS1 and miR-566, and the rescued effects of miR-566/FBXO47 on GABPB1-AS1 were evaluated. FBXO47 levels were increased by GABPB1-AS1 but decreased by miR-566 in A549 and H460 cells (Figs. 5A and 5B). As demonstrated by the western blot results, GABPB1-AS1 enhanced FBXO47 protein expression, but miR-566 reduced FBXO47 protein expression, which indicated the presence of a GABPB1-AS1/miR-566/FBXO47 pathway (Figs. 5C and 5D). Furthermore, based on functional analysis, the repressive effects on NSCLC cell proliferation (caused by lncRNA GABPB1-AS1) were attenuated by miR-566 overexpression (Figs. 5E and 5F). The migration and invasion assays using NSCLC cells yielded consistent results (Figs. 5G and 5J). Taking all data into consideration, we concluded that GABPB1-AS1 exerted its suppressive function on NSCLC cells through the miR-566/FBXO47 pathway.

### Overexpression of GABPB1-AS1 inhibited tumorigenesis in vivo

**Figure 6:**
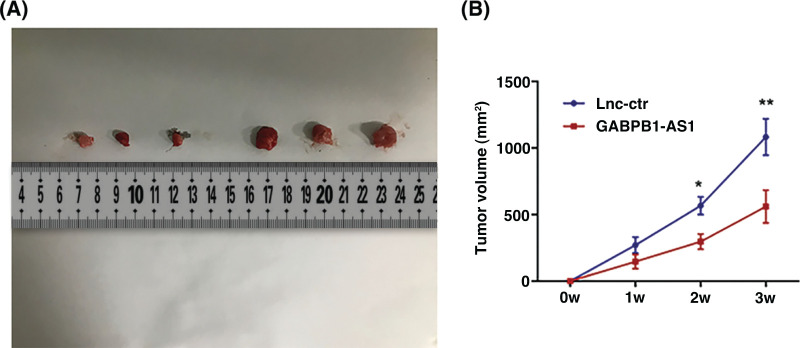
Overexpression of GABPB1-AS1 inhibited tumorigenesis *in vivo*. (A) Overexpression of GABPB1-AS1 significantly inhibited tumorigenesis in mice models. (B) The tumor volumes were measured and compared. *p < 0.05, **p < 0.01, n = 6.

To further verify the effects of GABPB1-AS1 *in vivo*, a total of 12 nude mice 6-week nude mice (NU/NU) were obtained from Vital River Inc. (Beijing, China). The 12 nude mice were randomly divided into the control group (n = 6) and the GABPB1-AS1 group (n = 6). After injection of NSCLC cells subcutaneously, we measured the tumor volume for three weeks. It was shown that overexpression of GABPB1-AS1 significantly inhibited tumorigenesis in mice models (Figs. 6A and 6B).

## Discussion

Increasing research reveal that some lncRNAs are involved in most physiological and pathological processes. Moreover, lncRNAs exert crucial impacts on cancer occurrence and development. It is wildly accepted that some lncRNAs control cancer cell proliferation, autophagy, apoptosis and metastasis. For example, lung cancer tissue samples expressed reduced lncRNA MIR22HG levels compared with normal lung tissues, and MIR22HG could accelerate lung cancer cell survival and cell death signaling by regulating the oncogenes YBX1, MET, and p21 [18]. LncRNA BLACAT2 is overexpressed in lymph node metastatic bladder cancer samples and correlated with clinical features. Bladder cancer-associated lymphangiogenesis and lymphatic metastasis are triggered by BLACAT2, which also induces VEGF-C expression [19]. Expression of lncRNA TSLNC8 was reduced in hepatocellular carcinoma (HCC) tissues and altered interleukin-6/STAT3-associated genes/pathways, cell proliferation and metastasis in HCC [20]. Therefore, it is reasonable for researchers to believe that some functional lncRNAs are essential to pathological processes in cancer cells.

The lncRNA GABPB1-AS1 was first identified by Tani et al. using 5′-Bromo-uridine immunoprecipitation chase-deep sequencing analysis [14]. Later, Qi et al. found that GABPB1-AS1 was upregulated by elastin and led to the downregulation of the gene encoding Peroxiredoxin-5 (PRDX5) peroxidase and then suppressed the cellular antioxidant capacity [21]. For its role in cancer, GABPB1-AS1 was found to be upregulated in glioma tissues and associated with advanced WHO grades [22]. It was also found that silencing of GABPB1-AS1 could significantly inhibit the proliferation, clonal formation, migration, and epithelial-mesenchymal transformation of osteosarcoma by acting the SP1/Wnt/β-catenin signaling pathway [23]. However, there is still no study investigated the specific role of GABPB1-AS1 in NSCLC. In this research, GABPB1-AS1’s expression pattern and biological role in NSCLC were firstly investigated. Reduced GABPB1-AS1 expression in tumor samples was observed. Moreover, by dividing the patients into two groups based on metastasis status, it was demonstrated that GABPB1-AS1 levels were considerably reduced in metastatic patients compared with non-metastatic patients. Hence, it is hypothesized that GABPB1-AS1 could play a role as a tumor suppressor in NSCLC. Phenotype analysis indicated that GABPB1-AS1 overexpression dramatically reduced NSCLC proliferation, migration and invasion. This is the first study to investigate GABPB1-AS1 expression and phenotypes in NSCLC. The anti-tumor character of GABPB1-AS1 was proposed in NSCLC, which was inconsistent with some former investigations of GABPB1-AS1 in other tumor types like glioma and osteosarcoma. This might be attributed to the heterogeneity, and lncRNA could play a different role in distinct cancer types.

One of the most important mechanisms of action of lncRNAs is binding to miRNA and acting as endogenous RNA (ceRNA). However, studies about GABPB1-AS1 are lacking, and its interaction with miRNA in NSCLC has not been previously explored. In the current study, an online bioinformatic algorithm was applied to predict GABPB1-AS1’s downstream molecules, and the prediction was confirmed by luciferase reporter assays. MiR-566 was predicted and verified as a direct target of GABPB1-AS1 in our current research. MiR-566 is also aberrantly expressed in some types of cancers. Global profiling from patients with lung adenocarcinoma demonstrated that miRNA-566 was one of the six miRNAs expressed at substantially higher levels in adenocarcinoma samples compared with control samples, potentially representing a diagnostic biomarker in the future [24]. RNA microarray and verification analysis by Drusco et al. demonstrated that miR-566 was downregulated in colon cancer metastatic samples, and the miRNAs signature could help to distinguish the sites of colorectal recurrences and metastatic or primary tumor [25]. Other studies also explored the impacts of this distinct miRNA and its downstream target gene. MiR-566 is overexpressed in human glioma cell and accelerates glioma cells proliferation and invasion as well as EGFR pathway activity. The silencing of miR-566 by lentivirus could sensitize glioblastoma cells to nimotuzumab [26]. Xiao et al. observed that miR-566 could directly target Hippel-Lindau, increase VEGF expression levels, and promote the invasive and migratory capacities of glioblastoma [27]. Notably, miR-566 was highly expressed in renal cell carcinoma and accelerated renal cell carcinoma cell proliferation and mobility. MiR-566 is a potential biomarker for the diagnosis and prognosis of renal cell carcinoma [28]. This research discovered and verified a new direct target of miR-566, FBXO47, in NSCLC cells. The targeted effects were verified from multiple angles. Moreover, our experiments further revealed that miR-566/FBXO47 could mediate the adverse effects of GABPB1-AS1 on NSCLC. How GABPB1-AS1 affects NSCLC cells may require future research in exploring downstream targets and signaling pathways of GABPB1-AS1/miR-566/FBXO47.

## Conclusions

In conclusion, lncRNA GABPB1-AS1 was downregulated significantly in NSCLC specimens and cell lines. The GABPB1-AS1 reduced NSCLC cell proliferation, migration and invasion by targeting miR-566/FBXO47. Our study may offer a better understanding of the occurrence and development of NSCLC and provide a potential target for future treatment.

## Data Availability

The data that supported the findings of this study are available on reasonable request from the corresponding author.
